# Barriers and facilitators to physical activity in people with hip or knee osteoarthritis: protocol for a systematic review of qualitative evidence

**DOI:** 10.1136/bmjopen-2016-012049

**Published:** 2016-11-03

**Authors:** Archontissa M Kanavaki, Alison Rushton, Rainer Klocke, Abhishek Abhishek, Joan L Duda

**Affiliations:** 1 School of Sport, Exercise and Rehabilitation Sciences, College of Life and Environmental Sciences, University of Birmingham, Birmingham, UK; 2 Department of Rheumatology, Dudley Group NHS Foundation Trust, Dudley, UK; 3 Faculty of Medicine and Health Sciences, Academic Rheumatology Unit, School of Medicine, University of Nottingham, Nottingham, UK

**Keywords:** knee osteoarthritis, hip osteoarthritis, physical activity, barriers, facilitators, systematic review protocol

## Abstract

**Introduction:**

This protocol aims to describe the objective and methods to be followed in a systematic review of qualitative studies on barriers and facilitators to physical activity (PA) in people with hip or knee osteoarthritis (OA).

**Methods and analysis:**

MEDLINE, EMBASE, PhychINFO, Web of Science, CINAHL, SPORTDiscus, Scopus and grey literature sources will be electronically searched. Hand search of qualitative research-centred journals, reference screening of relevant reviews and inquiries to researchers active in the field will complement the search. Studies will be selected if they apply qualitative or mixed-methods designs to directly explore factors that correspond to engagement in PA/exercise or, the perceptions regarding PA/exercise in people with hip or knee OA. The Critical Appraisal Skills Programme Qualitative Checklist and the evaluative criteria of credibility, transferability, dependability and confirmability will be applied for the study appraisal. 2 independent reviewers will perform the search, study selection and study appraisal. Thematic synthesis will be used for synthesising the findings of the primary studies and the process and product of the synthesis will be checked by a second researcher. ConQual approach will be used for assessing the confidence in the qualitative findings.

**Ethics and dissemination:**

This systematic review will inform our understanding of the PA determinants and how to optimise behaviour change in people living with hip or knee OA. The review findings will be reported in a peer-reviewed journal and presented at national or international conferences. The study raises no ethical issues.

**Trial registration number:**

CRD42016030024.

**Strengths and limitations of this study**

- To the best of our knowledge, this is the first systematic review of qualitative evidence on barriers and facilitators of physical activity (PA) in people with hip or knee osteoarthritis (OA). Further, differences in barriers and facilitators between (1) exercise and lifestyle PA, and (2) uptake and maintenance of PA will be explored. This will largely contribute to our understanding of PA behaviours and provide information on how to optimise behaviour change in the population of interest.
- Rigorous methods will be applied informed by the Centre for Reviews and Dissemination and Cochrane Qualitative Research Methods Group guidelines and reported at all stages in line with the Enhancing Transparency in Reporting the Synthesis of Qualitative Research (ENTREQ) statement.
- The level of confidence in each review finding will be reported.
- One limitation of this systematic review is that only papers written in English will be included.

## Introduction

### Rationale

Osteoarthritis (OA) is a common joint disease and one of the main causes of disability in ageing populations.^1^ Physical activity (PA) has a key role in the management of OA. For instance, exercise, which is a structured and purposeful form of PA^2^ is effective in reducing pain and improving physical function and health-related quality of life in knee and hip OA.^3–8^ In addition, sedentary pursuits have been linked to a decline in physical function irrespective of the time the patients spent in moderate-to-vigorous activities.^9^ Maintaining a physically active lifestyle (ie, time spent in leisure and non-leisure physical activities, not limited to engagement in exercise) is therefore important for people living with lower limb OA.^1^ Nonetheless, the majority of people with knee or hip OA do not meet the guideline recommendations of at least 150 min of moderate-to-vigorous physical activity per week and are reported to be less physically active than their counterparts without OA.^10^ ^11^ Furthermore, in the case of existing exercise interventions in this population, PA maintenance postintervention is a major issue.^12^ ^13^

An emerging question is therefore what are the PA determinants in people with hip or knee OA, so that they can be optimally applied in healthcare practice and policymaking to improve health outcomes. Existing narrative^14^ ^15^ and systematic^16^ ^17^ reviews have addressed this question. In the most up-to-date quantitative systematic review of factors influencing PA in this population,^16^ demographic characteristics, physical function and symptom severity were the only PA correlates consistently reported by the studies. There was inconsistent association with psychological factors like mental health. The paucity of studies on social and environmental correlates of PA was highlighted in this review.^16^ When it comes to understanding behaviour and behaviour change though, personal (eg, cognitions, attitudes), as well as social and environmental factors are of major importance.^18–21^

To date, no systematic work has captured these factors, with those identified which are modifiable potentially contributing to the development of interventions to promote the initiation and maintenance of PA in people with OA. Qualitative studies, which offer an in-depth exploration of the human experience, might prove more appropriate in illustrating the variety and interplay of psychosocial and environmental factors that facilitate or hinder PA specifically in people living with lower limb OA. A recent scoping review of quantitative and qualitative studies^22^ has mapped modifiable factors linked to exercise participation in patients with hip and knee OA using the Theoretical Domains Framework. This systematic review of qualitative evidence will move one step further by applying rigorous methodology, such as quality appraisal of the included studies and confidence in the reported findings. Confidence in the reported findings is directly relevant to how informative and useful they can be in practice. In addition, two important distinctions of potential relevance to barriers and facilitators to PA will also be addressed in this systematic review. The first is a discrimination between barriers and facilitators to exercise and ‘lifestyle’ PA. The second is about the theoretical and empirical distinction between uptake and maintenance of PA, that is, whether PA is a newly introduced or reintroduced behaviour in a person's life or its regular engagement is part of one's lifestyle.^23^ Different factors can act as barriers and facilitators at different stages of behavioural change (in particular, when the focus is on adoption or maintenance), which holds practical implications when it comes to identifying key elements of behavioural interventions.

### Objectives

To identify, appraise and synthesise the existing qualitative evidence on barriers and facilitators to PA uptake and/or maintenance in people with hip or knee OA based on the patients' perceptions and experiences.

Secondary objectives are to explore differences in barriers and facilitators between (1) exercise and lifestyle PA and (2) uptake and maintenance of PA.

## Methods

This systematic review protocol follows the Preferred Reporting Items for Systematic Review and Meta-analysis Protocols (PRISMA-P) 2015 statement (see online supplementary appendix 1).^24^ ^25^ The systematic review was registered with the International Prospective Register of Systematic Reviews (PROSPERO), registration number CRD42016030024. It will be informed by the Centre for Reviews and Dissemination^26^ and Cochrane Qualitative Research Methods Group^27^ ^28^ guidelines and will follow the Enhancing Transparency in Reporting the Synthesis of Qualitative Research (ENTREQ)^29^ and the PRISMA^30^ statements for reporting systematic reviews (see online supplementary appendix 2). In the case of sections applicable to qualitative systematic reviews that are included in PRISMA, but are not covered by ENTREQ, these will also be reported.

10.1136/bmjopen-2016-012049.supp1 supplementary appendix

10.1136/bmjopen-2016-012049.supp2 supplementary appendix

### Eligibility criteria

The criteria outlined below will be used for study selection (see online supplementary appendix 3). PICOS (Population, Intervention, Comparison, Outcomes, Study design), which is an established tool for defining key components of research questions,^31^ was adapted for use in this study. In particular, interventions and comparators were not applicable and the phenomenon of interest will be identified instead.

10.1136/bmjopen-2016-012049.supp3 supplementary appendix

*Population*: Study participants will be adults who have physician-diagnosed hip or knee OA; or radiographic OA using Kellgren and Lawrence grade ≥2 at hip or knee; or meet internationally accepted classification criteria for OA (eg, American College of Rheumatology classification criteria). If the study population involves groups of patients with other types of arthritis, for example, rheumatoid arthritis, they will be included in this study provided that patients with knee and hip OA combined are the highest proportion of participants. Studies will be excluded if the study participants are people about to undergo or have undergone total hip or knee arthroplasty.

*Outcomes* will be barriers and facilitators that influence uptake and/or maintenance of PA in people with OA as perceived and reported by the patients.

Studies will be included if (1) they directly explore the factors/barriers/facilitators/motivation that correspond to engagement in PA/exercise (ie, this is stated in the study objectives or relevant interview questions are included); or (2) they directly address or focus on any aspect of the experience or perceptions of people living with hip or knee OA regarding PA and/or exercise.

*Study designs*: (1) Qualitative studies using appropriate methods of data collection and data analysis. (2) Mixed methods studies that report qualitative findings.

*Language* studies will be excluded if written in a language other than English.

*Publication year*: From database inception to 31 December 2015.

### Information sources

The databases MEDLINE (Ovid MEDLINE(R) In-Process and Other Non-Indexed Citations and Ovid MEDLINE(R) 1946 to Present, OVID interface), EMBASE (1974 onwards, OVID interface), PhychINFO (1967 onwards, OVID interface), Web of Science, CINAHL, SPORTDiscus and Scopus will be searched from inception to 31 December 2015. Also, grey literature sources will be considered, that is, OpenGrey, National Health Service (NHS) evidence. Hand search of qualitative research-centred journals, for example, *Qualitative Health Research*, *Sociology of Health and Illness*, will complement the search strategy. Screening of the references of included articles and relevant existing reviews will take place. Finally, active researchers in the field who have contributed to this literature will be contacted.

### Search

The search strategy will comprise comprehensive keyword combinations for each of the four concepts of interest (see online supplementary appendix 4 for MEDLINE), that is, (1) knee and hip OA (1–9 in the online supplementary appendix), (2) PA/exercise (10–16), (3) barriers, facilitators, motivation, uptake, maintenance (17–24), (4) qualitative study design (25–30). Free-text search (.mp) will be applied for the basic search terms for each concept (eg, ‘osteoarthritis’ for population; ‘physical activity’, ‘exercise’ for phenomenon of interest; ‘barrier*’, ‘facilitator*’, ‘motivation’ for outcomes; ‘qualitative’ for study design), supplemented by a wide array of alternative terms searched for in the title/abstract section or free-text search. Within each group of concepts, the keyword combinations will be mutually inclusive (‘OR’ operator). The combination of the four groups was applied in the latter stage using the AND operator.

10.1136/bmjopen-2016-012049.supp4 supplementary appendix

### Study records

The study selection process will be according to the PRISMA flow diagram^30^ (see online supplementary appendix 5). Two independent reviewers will run the search and study selection. Endnote X7 software will be used for data management. Citations including abstracts will be imported and duplicates will be removed. Selected articles will be juxtaposed for multiple reports of a single study, so that double counting of studies is avoided.

10.1136/bmjopen-2016-012049.supp5 supplementary appendix

The predetermined eligibility criteria will be used in the form of a list (see online supplementary appendix 3), which will be checked and fine-tuned if necessary by the two reviewers. The reviewers will independently apply the criteria at all stages of the selection process. After title/abstract screening, full-text copies of potentially relevant studies will be obtained. Additional information will be sought from authors if necessary at the stage of full-text assessment. Where the information provided is insufficient for study selection, assessment and synthesis, the respective studies will not be included in the synthesis but will be referenced in the discussion section. Consensus will be reached through discussion and where agreement is not reached, a third reviewer will be consulted. At the end of the selection process, the κ statistic^32^ will be used to assess the chance-corrected agreement between the reviewers in assessing the full-text articles as included, excluded or unclear. A supplementary table with information about the selected studies will be provided including study characteristics (first author's name, publication year, method of data collection and data analysis), participant characteristics (age, gender, locus and severity of OA, duration of diagnosis, physical activity profile) and contextual information (country, geographic area, setting if applicable). Data will be entered in and managed with NVivo V.11 qualitative data analysis software (QSR International).

### Data items

All text under the sections of ‘results’ and ‘findings’ will be considered as data and will be analysed. If findings and discussion are presented together, then discussion will also be considered as a data item.

## Outcomes and prioritisation

### Phenomenon of interest

The description and interpretation of patients with OA experiences and perceptions regarding what hinders and what facilitates and motivates them to engage in PA behaviours constitute the phenomenon of interest. All types of factors reported by the participants will be included, for example, health-related, psychological, social, cultural, environmental. Subgroups of the phenomenon of interest will also be explored, provided that there is sufficient evidence. These are: barriers and facilitators to PA uptake and PA maintenance; engagement in exercise and engagement in lifestyle PA.

### Appraisal of study quality

Since there is no consensus on how to assess qualitative evidence and a single set of criteria might not be applicable to all kinds of qualitative research,^33^ ^34^ two different approaches to appraisal will be applied (see online supplementary appendix 6).

10.1136/bmjopen-2016-012049.supp6 supplementary appendix

First the Critical Appraisal Skills Programme (CASP) Qualitative Checklist, a structured tool commonly employed in systematic reviews (SRs) of qualitative evidence, will be used. CASP Qualitative Checklist is broadly suitable for various qualitative study designs, is available online and free of charge. The tool, including introduction, 10 questions and prompts, will be used as provided by the CASP-uk.net. Studies will be rated as ‘high quality’ if they meet at least 8 of the 10 criteria, ‘medium quality’ if they meet 5–7 of the criteria and ‘low quality’ if they meet 4 or less.

Although the CASP tool appraises reporting and methodological quality, it does not address aspects of the research validity^35^ and can favour papers that are less insightful as long as they comply with ‘expectations of research practice’.^36^ To address this gap, the evaluative criteria of credibility, transferability, dependability and confirmability^37^ will be applied. These criteria widely acknowledge the philosophical stance of qualitative research, focus on the trustworthiness of the study^37^ ^38^ and their development was not aimed in particular at the evaluation of interpretive qualitative approaches as other theoretically informed tools, for example, Popay *et al*.^39^ Included studies will be assessed as to whether they apply the techniques suggested for ensuring study quality according to Lincoln and Guba's criteria:^33^ ^40^ prolonged engagement, persistent observation, peer review, triangulation, negative case analysis, referential adequacy and member checking to ensure credibility; thick description for transferability; inquiry audit for dependability; confirmability audit, audit trail, triangulation and reflexivity to ensure confirmability. A more detailed description of the context of the above procedures can be found in online supplementary appendix 6. Studies will be rated as ‘high quality’ if they meet at least three of the four criteria, ‘medium quality’ if they meet two of the criteria and ‘low quality’ if they meet one or none.

Two reviewers, both with qualitative research training and experience (AMK/NE) and one with additional experience in qualitative systematic reviews (NE), will independently appraise the selected studies. First, the appraisal process will be piloted, that is, the reviewers will independently apply the two sets of criteria on two studies and criteria and then compare the outcome and discuss the process they followed, so that potential discrepancies in applying the criteria are resolved. The final assessment for each study will be reached through discussion and in case a consensus is not reached, a third researcher will be consulted. A detailed justification of the assessment outcome for the second set of criteria will be available on publication of the SR.

### Data synthesis

Thematic synthesis as described by Thomas and Harden^41^ will be applied for data synthesis. Thematic synthesis is a transparent and suitable method for integrating qualitative evidence in a SR and has been used for SRs of barriers and facilitators to various behaviours.^42–44^ The synthesis involves three stages: (1) free line-by-line coding; (2) grouping of the codes into ‘descriptive themes’, which also includes the translation of conceptions from one study to the other (ie, the codes from all included studies will be compared with each other in an iterative process, the codes/quotes describing the same concept will be merged under one code and those expressing a similar concept will be grouped together); and (3) the formation of analytical themes. At the latter stage, barriers and facilitators to PA in people with hip and knee OA will be inferred from the descriptive themes; that is, the research questions, which are put aside during the data-driven first two stages, will be introduced at this point to inform the formation of analytical themes. Therefore, the synthesis will combine both an inductive (at first stages) and a deductive (latter stage) approach. The analytical themes and their relation with descriptive themes will be presented in tables. The synthesis will be conducted by one researcher (AMK) and checked by a second independent reviewer with experience in thematic analysis (NE), to enhance credibility.

### Confidence in the synthesised qualitative findings

Assessing the quality of the studies in a SR does not answer the question of how much certainty or trust we can place on each individual review finding. To ensure the potential value of the review in informing its users, the assessment of the trust that can be placed on each individual finding is advised.^45^ In qualitative evidence syntheses, approaches to confidence in the findings have only recently been developed.^38^ ^46^ The ConQual approach,^38^ which was developed by qualitative research experts from the Joanna Briggs Institute in Adelaide, will be adopted for assessing the confidence in the findings. ConQual assesses the confidence in findings, that is, truth value, based on two elements: dependability and credibility (see online supplementary appendix 7). ConQual is the approach of choice as it offers a clear operationalisation of each element and description of the appraisal process. A Confidence in the Findings Table will be formulated which will include the review finding, the assessments for dependability, credibility and the overall confidence score (high, moderate, low, very low).

10.1136/bmjopen-2016-012049.supp7 supplementary appendix

## Discussion

This systematic review will be the first to synthesise and report barriers and facilitators of PA in people with hip or knee OA based on qualitative evidence. Following the emerging evidence on the independent role of sedentary pursuits on health and mortality^47^ ^48^ and the shifting of health guidelines and policies from exercise promotion to physical activity promotion, we will further explore differences between determinants of lifestyle PA and exercise, as there is a pronounced gap in the literature regarding the former.^49^ Additionally, we will explore differences reported in the literature between uptake and maintenance of PA. The review findings will inform our understanding of factors facilitating or inhibiting participation in physical activity and provide information on how to optimise behaviour change at different stages (ie, uptake or maintenance) in the targeted population.

This protocol serves to provide a detailed account of the rational and methods to be used in the proposed systematic review to ensure the transparency of the process.^24^ In case any deviation from the protocol takes place, it will be justified and discussed in the systematic review on publication.
