# Supplementary material for: Barriers and facilitators to physical activity in people with hip or knee osteoarthritis: protocol for a systematic review of qualitative evidence
Source: BMJ Open. 2016 Nov 3;6(11):e012049. doi: 10.1136/bmjopen-2016-012049 (PMC5128852; doi:10.1136/bmjopen-2016-012049)
Supplement: supplementary appendix [file bmjopen-2016-012049supp_appendix2.pdf]

Appendix 2.

ENTREQ Statement: Recommended items to address in a synthesis of qualitative research

No	Item	Guide and description
1	Aim	State the research question the synthesis addresses.
2	Synthesis methodology	Identify the synthesis methodology or theoretical framework which underpins the synthesis, and describe the rationale for choice of methodology.
3	Approach to searching	Indicate whether the search was pre-planned or iterative.
4	Inclusion criteria	Specify the inclusion/exclusion criteria.
5	Data sources	Describe the information sources used and when the searches conducted; provide the rationale for using the data sources.
6	Electronic Search strategy	Describe the literature search.
7	Study screening methods	Describe the process of study screening and sifting.
8	Study characteristics	Present the characteristics of the included studies.
9	Study selection results	Identify the number of studies screened and provide reasons for study exclusion.
10	Rationale for appraisal	Describe the rationale and approach used to appraise the included studies or selected findings.
11	Appraisal items	State the tools, frameworks and criteria used to appraise the studies or selected findings.
12	Appraisal process	Indicate whether the appraisal was conducted independently by more than one reviewer and if consensus was required.
13	Appraisal results	Present results of the quality assessment and indicate which articles, if any, were weighted/excluded based on the assessment and give the rationale.
14	Data extraction	Indicate which sections of the primary studies were analysed and how were the data extracted from the primary studies.
15	Software	State the computer software used, if any.

**16**	Number of reviewers	Identify who was involved in coding and analysis.
**17**	Coding	Describe the process for coding of data.
**18**	Study comparison	Describe how were comparisons made within and across studies.
**19**	Derivation of themes	Explain whether the process of deriving the themes or constructs was inductive or deductive.
**20**	Quotations	Provide quotations from the primary studies to illustrate themes/constructs, and identify whether the quotations were participant quotations of the author's interpretation.
**21**	Synthesis output	Present rich, compelling and useful results that go beyond a summary of the primary studies.

From: Tong A, Flemming K, McInnes E, Oliver S, Craig .(2012). Enhancing transparency in reporting the synthesis of qualitative research: ENTREQ. BMC Medical Research Methodology, 12(1):181.

PRISMA Statement: Recommended items to address in Systematic Reviews and Meta-Analyses

Section/topic	#	Checklist item	Reported on page #
**TITLE**			
Title	1	Identify the report as a systematic review, meta-analysis, or both.	
**ABSTRACT**			
Structured summary	2	Provide a structured summary including, as applicable: background; objectives; data sources; study eligibility criteria, participants, and interventions; study appraisal and synthesis methods; results; limitations; conclusions and implications of key findings; systematic review registration number.	
**INTRODUCTION**			
Rationale	3	Describe the rationale for the review in the context of what is already known.	
Objectives	4	Provide an explicit statement of questions being addressed with reference to participants, interventions, comparisons, outcomes, and study design (PICOS).	
**METHODS**			
Protocol and registration	5	Indicate if a review protocol exists, if and where it can be accessed (e.g., Web address), and, if available, provide registration information including registration number.	
Eligibility criteria	6	Specify study characteristics (e.g., PICOS, length of follow-up) and report characteristics (e.g., years considered, language, publication status) used as criteria for eligibility, giving rationale.	
Information sources	7	Describe all information sources (e.g., databases with dates of coverage, contact with study authors to identify additional studies) in the search and date last searched.	

Search	8	Present full electronic search strategy for at least one database, including any limits used, such that it could be repeated.	
Study selection	9	State the process for selecting studies (i.e., screening, eligibility, included in systematic review, and, if applicable, included in the meta-analysis).	
Data collection process	10	Describe method of data extraction from reports (e.g., piloted forms, independently, in duplicate) and any processes for obtaining and confirming data from investigators.	
Data items	11	List and define all variables for which data were sought (e.g., PICOS, funding sources) and any assumptions and simplifications made.	
Risk of bias in individual studies	12	Describe methods used for assessing risk of bias of individual studies (including specification of whether this was done at the study or outcome level), and how this information is to be used in any data synthesis.	
Summary measures	13	State the principal summary measures (e.g., risk ratio, difference in means).	
Synthesis of results	14	Describe the methods of handling data and combining results of studies, if done, including measures of consistency (e.g., I^2) for each meta-analysis.	
Risk of bias across studies	15	Specify any assessment of risk of bias that may affect the cumulative evidence (e.g., publication bias, selective reporting within studies).	
Additional analyses	16	Describe methods of additional analyses (e.g., sensitivity or subgroup analyses, meta-regression), if done, indicating which were pre-specified.	
**RESULTS**			
Study selection	17	Give numbers of studies screened, assessed for eligibility, and included in the review, with reasons for exclusions at each stage, ideally with a flow diagram.	
Study characteristics	18	For each study, present characteristics for which data were extracted (e.g., study size, PICOS, follow-up period) and provide the citations.	
Risk of bias within studies	19	Present data on risk of bias of each study and, if available, any outcome level assessment (see item 12).	
Results of individual studies	20	For all outcomes considered (benefits or harms), present, for each study: (a) simple summary data for each intervention group (b) effect estimates and confidence intervals, ideally with a forest plot.	
Synthesis of results	21	Present the main results of the review. If meta-analyses are done, include for each, confidence intervals and measures of consistency.	
Risk of bias across studies	22	Present results of any assessment of risk of bias across studies (see Item 15).	
Additional analysis	23	Give results of additional analyses, if done (e.g., sensitivity or subgroup analyses, meta-regression [see Item 16]).	
**DISCUSSION**			
Summary of evidence	24	Summarize the main findings including the strength of evidence for each main outcome; consider their relevance to key groups (e.g., healthcare providers, users, and policy makers).	

Limitations	25	Discuss limitations at study and outcome level (e.g., risk of bias), and at review-level (e.g., incomplete retrieval of identified research, reporting bias).	
Conclusions	26	Provide a general interpretation of the results in the context of other evidence, and implications for future research.	
**FUNDING**			
Funding	27	Describe sources of funding for the systematic review and other support (e.g., supply of data); role of funders for the systematic review.	

From: Moher D, Liberati A, Tetzlaff J, Altman DG, The PRISMA Group (2009). Preferred Reporting Items for Systematic Reviews and Meta-Analyses: The PRISMA Statement. PLoS Med 6(7): e1000097. doi:10.1371/journal.pmed1000097
