# Supplementary material for: Barriers and facilitators to physical activity in people with hip or knee osteoarthritis: protocol for a systematic review of qualitative evidence
Source: BMJ Open. 2016 Nov 3;6(11):e012049. doi: 10.1136/bmjopen-2016-012049 (PMC5128852; doi:10.1136/bmjopen-2016-012049)
Supplement: supplementary appendix [file bmjopen-2016-012049supp_appendix3.pdf]

Appendix 3. Eligibility criteria

Title:.<br>………<br>……<br><br>Author(s) and date: ……<br><br>*Study should be deemed eligible if responses to all items are under the "yes" column.*		
	**Yes**	**No**
1. Qualitative study design or mixed methods design.		
2. Participants are adults with a physician's diagnosis of hip or knee osteoarthritis, regardless of radiographic evidence. If the study sample also involves groups of patients with other types of arthritis, then the group with the highest proportion of patients should be that of knee and/ or hip OA.		
3. (a) The study directly (i.e. it is stated so in the study aims or, relevant interview questions are included) explores the factors/ barriers/ enablers/motivation that correspond to engagement/ adoption/ maintenance of PA/ exercise. Or (b) the study directly addresses or focuses on any aspect of the experience or perceptions of people living with hip or knee OA regarding PA and/ or exercise.		
4. Participants have not undergone and are not about to undergo hip or knee arthroplasty.		
5. Written in English.		
