# Supplementary material for: Barriers and facilitators to physical activity in people with hip or knee osteoarthritis: protocol for a systematic review of qualitative evidence
Source: BMJ Open. 2016 Nov 3;6(11):e012049. doi: 10.1136/bmjopen-2016-012049 (PMC5128852; doi:10.1136/bmjopen-2016-012049)
Supplement: supplementary appendix [file bmjopen-2016-012049supp_appendix4.pdf]

Appendix 4. Medline Search Strategy

Draft MEDLINE search- Ovid interface

1	osteoarthritis.mp. or exp Osteoarthritis, Hip/ or exp Osteoarthritis/ or exp Osteoarthritis, Knee/
2	(osteoarthriti* or osteo-arthriti* or osteoarthrotic or osteoarthros*).ti,ab.
3	(coxarthrosis or gonarthrosis).ti,ab.
4	"knee pain".mp.
5	"hip pain".mp.
6	"lower limb".mp.
7	exp Lower Extremity/ or "lower extremit*".mp.
8	(degenerative adj2 arthritis).ti,ab.
9	1 or 2 or 3 or 4 or 5 or 6 or 7 or 8
10	physical activity.mp. or exp Motor Activity/
11	exp Exercise/ or exp Exercise Therapy/ or exercise.mp.
12	exp Sports/ or sports.mp.
13	exp Life Style/ or exp Sedentary Lifestyle/ or sedentary.mp.
14	"non-exercis*".ti,ab.
15	"activities of daily living".mp. or exp "Activities of Daily Living"/
16	10 or 11 or 12 or 13 or 14 or 15
17	(maintain* or maintenance or support* or ongoing or "on-going" or adherence or reinforc* or comply* or compliance or "long-term" or adoption or engagement or avoidance or boost* or refresh* or remind* or promotion or promot* or "physical activity uptake" or "behavio* change" or "lifestyle change").ti,ab.
18	(barrier* or impediment or limit* or facilitator* or enablers or enabl* or motivators or motivat* or influenc* or factors or determinants).ti,ab.
19	facilitator*.mp.
20	barrier*.mp.
21	adherence.mp.
22	exp Motivation/ or motivators.mp.
23	social support.mp. or exp Social Support/
24	17 or 18 or 19 or 20 or 21 or 22 or 23
25	exp Qualitative Research/ or qualitative.mp.
26	(interview* or theme* or experience).mp. [mp=title, abstract, original title, name of substance word, subject heading word, keyword heading word, protocol supplementary concept word, rare disease supplementary concept word, unique identifier]
27	("content analysis" or "grounded theory" or "thematic analysis" or "phenomenological analysis" or phenomenolog* or narrative* or discourse or ethnograph*).ti,ab.
28	(("semi-structured" or semistructured or unstructured or informal or "in-depth" or indepth or "face-to-face" or structured or guide) adj3 (interview* or discussion* or questionnaire*)).ti,ab.

29	(focus group* or interview* or fieldwork or "field work" or triangulation or "data saturation" or "key informant").ti,ab.
30	25 or 26 or 27 or 28 or 29
31	9 and 16 and 24 and 30
