# Supplementary material for: Barriers and facilitators to physical activity in people with hip or knee osteoarthritis: protocol for a systematic review of qualitative evidence
Source: BMJ Open. 2016 Nov 3;6(11):e012049. doi: 10.1136/bmjopen-2016-012049 (PMC5128852; doi:10.1136/bmjopen-2016-012049)
Supplement: supplementary appendix [file bmjopen-2016-012049supp_appendix6.pdf]

Appendix 6. Quality appraisal: CASP Qualitative Checklist and Evaluative criteria for Trustworthiness.

Title: ………………………………………………………………………………………<br>Author(s) and date: ………………………………………………………………………<br>Study No: ……………………………………………………………………………………….<br><br>**Critical Appraisal Skills Programme Qualitative Checklist.**	**Yes**	**No**	**Can't answer**
1. Was there a clear statement of the aims of the research?<br>*What was the goal of the research? Why it was thought important?*			
2. Is a qualitative methodology appropriate?<br>*If the research seeks to interpret or illuminate the actions and/or subjective experiences of research participants. Is qualitative research the right methodology for addressing the research goal?*			
3. Was the research design appropriate to address the aims of the research?<br>*If the researcher has justified the research design (e.g. have they discussed how they decided which method to use)?*			
4. Was the recruitment strategy appropriate to the aims of the research?<br>*If the researcher has explained how the participants were selected. If they explained why the participants they selected were the most appropriate to provide access to the type of knowledge sought by the study. If there are any discussions around recruitment (e.g. why some people chose not to take part).*			
5. Was the data collected in a way that addressed the research issue?<br>*If the setting for data collection was justified. If the researcher has justified the methods chosen. If the researcher has made the methods explicit (e.g. for interview method, is there an indication of how interviews were conducted, or did they use a topic guide)? If the methods were modified during the study. If so, has the researcher explained how and why? If the form of data is clear (e.g. tape recordings, video material, notes etc). If the form of data is clear (e.g. tape recordings, video material, notes etc). if the researcher has discussed saturation of data.*			
6. Has the relationship between researcher and participants been adequately considered?<br>*If the researcher critically examined their own role, potential bias and influence during (a) Formulation of the research questions (b) Data collection, including sample recruitment and choice of location How the researcher responded to events during the study and whether they considered the implications of any changes in the research design.*			
7. Have ethical issues been taken into consideration?<br>*If there are sufficient details of how the research was explained to participants for the reader to assess whether ethical standards were maintained If the researcher has discussed issues raised by the study (e.g. issues around informed consent or confidentiality or how they have handled the effects of the study on the participants during and after the study). If approval has been soughtfrom the ethics committee.*			
8. Was the data analysis sufficiently rigorous?<br>*If there is an in-depth description of the analysis process If thematic analysis is used. If so, is it clear how the categories/themes were derived from the data? Whether the researcher explains how the data presented were selected from the original sample to demonstrate the analysis process. If sufficient data are presented to support the findings. To what extent contradictory data are taken into account. Whether the researcher critically examined their own role, potential bias and influence during analysis and selection of data for presentation.*			
9. Is there a clear statement of findings?<br>*If the findings are explicit If there is adequate discussion of the evidence both for and against the researchers' arguments. If the researcher has discussed the credibility of their findings (e.g. triangulation, respondent validation, more than one analyst). If the findings are discussed in relation to the original research question.*			
10. How valuable is the research?<br>*If the researcher discusses the contribution the study makes to existing knowledge or understanding e.g. do they consider the findings in relation to current practice or policy or relevant research-based literature? If they identify new areas where research is necessary If the researchers have discussed*			

whether or how the findings can be transferred to other populations or considered other ways the research may be used.			

**Criteria for trustworthiness based on Creswell (2007) and Cohen & Crabtree (2006)**	Reviewer's assessment (Technique applied? How?)
**Credibility**	
**Prolonged engagement and persistent observation.** Do the researchers spend sufficient time in the field, observe, talk to different people, build relationships, check for misinformation stemming from the researcher or the informants?	
**Triangulation.** Do the researchers make use of multiple data sources, investigators, theories to enhance understanding and ensure a rich and robust account of the study inquiry?	
**Peer review or debriefing.** "External check of the research process" (Creswell, 2007; p.208) or exposition of the research process to an unaffected peer. Do sessions between the researcher and a peer take place? Are written accounts of these sessions being kept?	
**Negative case analysis**. Do the researchers take account of the data that do not fit with emerging patterns or explanations? Do they revise the initial hypotheses and analysis until it accounts for the majority of cases?	
**Referential adequacy.** "Identifying a portion of data to be archived, but not analysed. The researcher then conducts the data analysis on the remaining data and develops preliminary findings. The researcher then returns to this archived data and analyses it as a way to test the validity of his or her findings" (Cohen & Crabtree, 2006).	
**Member checking**. Do the researchers take data, analyses, interpretations, conclusions back to the participants to evaluate the truthfulness of the account?	
**Transferability**	
**Thick description** refers to "describing and interpreting observed social action (or behaviour) within its particular context" (Ponterotto, 2006) Does the author achieve to give a sense of verisimilitude? Does the author describe in detail each part of the study (fully describing the study participants; settings and procedures, such as location and length of the interviews, recording procedures, interviewer's and interviewee's reactions; results, e.g. long quotes from the participants or the interview dialogue; successfully bringing together the participants' experiences with the researchers' interpretation of those in discussion)?	
**Dependability**	
**External audit.** ("Inquiry audit") Is there an "external consultant", who is not part of the study, examining the process and product of the study?	
**Confirmability**	
**External audit** ("confirmability audit")	
**Reflexivity.** (Clarification of researcher bias) Are the authors reflexive, i.e. do they "identify the perspectives they bring to their studies as insiders and/ or outsiders" and ways through which those affect "how they analyse, interpret and report the findings" (Sparkes & Smith, 2014: p 181-3). Is there a "critical friend" to help in this process?	
**Triangulation** (as above)	
**Audit trail.** Is the process of the study transparent and trackable? Do the researchers provide descriptions of the decision making process in detail?	
