# Supplementary material for: Barriers and facilitators to physical activity in people with hip or knee osteoarthritis: protocol for a systematic review of qualitative evidence
Source: BMJ Open. 2016 Nov 3;6(11):e012049. doi: 10.1136/bmjopen-2016-012049 (PMC5128852; doi:10.1136/bmjopen-2016-012049)
Supplement: supplementary appendix [file bmjopen-2016-012049supp_appendix7.pdf]

Appendix 7. ConQual criteria for assessing Confidence in the synthesised findings

Dependability. *When the five criteria for dependability are not met across the included studies the synthesised finding is downgraded based on the aggregated level of dependability.*
1. Is there congruity between the research methodology and the research question or objectives?<br>2. Is there congruity between the research methodology and the methods used to collect data?<br>3. Is there congruity between the research methodology and the representation and analysis of data?<br>4. Is there a statement locating the researcher culturally or theoretically?<br>5. Is the influence of the researcher on the research, andvice-versa, addressed?
Credibility. *When not all the findings included in a synthesised finding are considered unequivocal downgrading may occur.*
Unequivocal (findings accompanied by an illustration that is beyond reasonable doubt and; therefore not open to challenge).<br>Equivocal (findings accompanied by an illustration lacking clear association with it and therefore open to challenge).<br>Unsupported (findings are not supported by the data).

*From: Munn Z, Porritt K, Lockwood C, et al. Establishing confidence in the output of qualitative research synthesis: the ConQual approach. BMC Medical Research Methodology 2014;**14**(1):1-7.*
